# Abdominal lymph node metastasis in non-surgical esophageal squamous cell carcinoma: prognostic significance and a novel staging strategy

**DOI:** 10.3389/fonc.2023.1234426

**Published:** 2023-10-09

**Authors:** Zongxing Zhao, Hongmin Wang, Yajing Liu, Minghuan Li, Mingjun Li

**Affiliations:** ^1^Department of Radiation Oncology, Liaocheng People’s Hospital, Shandong First Medical University, Liaocheng, Shandong, China; ^2^Joint Laboratory for Translational Medicine Research, Liaocheng People’s Hospital, Shandong First Medical University, Liaocheng, Shandong, China; ^3^Clinical Laboratory, Liaocheng Third People’s Hospital, Liaocheng, Shandong, China; ^4^Department of Radiation Oncology, Shandong Cancer Hospital, Shandong First Medical University, Jinan, Shandong, China

**Keywords:** lymph node metastases, ESCC (esophageal squamous cell carcinoma), stage, radiation, prognosis

## Abstract

**Purpose:**

This study aimed to evaluate the feasibility of a combination of abdominal lymph node (LN) metastasis and the number of LNs in esophageal squamous cell carcinoma (ESCC) patients to optimize its clinical nodal staging.

**Methods:**

A retrospective study, including a total of 707 ESCC patients treated with definitive radiotherapy, was conducted at two participating institutes. Different combinations of LN variables, including abdominal LN metastasis (R1: no-abdominal LN metastasis; R2: abdominal LN metastasis), were further analyzed to propose a potential revised nodal (rN) staging.

**Results:**

The multivariate analyses showed that the number of metastatic LN and abdominal LN metastasis were independent prognostic factors for the overall survival (OS). The results showed no significant differences in the OS between the N2 patients with abdominal LN metastasis and N3 patients. The OS of the stage III patients with abdominal LN metastasis was not significantly different from those with stage IVa. The N3R1 and N1-2R2 had similar hazard ratios (HRs). The N1R1 subset was defined as rN1, the N2R1 subset was defined as rN2, and the N3R1-2 and N1-2R2 subsets were defined as rN3. The HRs of OS of the rN2 and rN3 groups increased subsequently. The rN stage could identify the differences in the OS times of each subgroup based on the 8th AJCC cN staging or the 11th JES N staging.

**Conclusions:**

The rN staging, including the number of metastatic LNs and abdominal LN metastasis, might serve as a potential prognostic predictor for non-surgical patients with ESCC.

## Introduction

Esophageal squamous cell carcinoma (ESCC) occurs more frequently in Asian countries (1). Neoadjuvant chemoradiotherapy (CRT) followed by surgery could improve the R0 resection and survival of the locally advanced ESCC patients compared to surgery alone (2). The combined modality treatment, including radiotherapy and concurrent chemotherapy, is used for non-surgical ESCC patients. Currently, the 5-year survival rate of ESCC patients treated with CRT is 10%–30% (3–5). Lymph node (LN) metastasis is an important prognostic factor of ESCC. According to the latest edition of the American Joint Committee on Cancer (AJCC) staging, among the Tumor–Node–Metastasis (TNM) stages of ESCC, the N stage is determined by the number of LNs (6). However, numerous reports showed that the N stage could not be determined only by the number of LNs (7–10). Studies focused on the correlations between the prognosis of ESCC with other node variables (11–14). Unfortunately, the differences in treatment methods and pathological types have resulted in a wide diversity in prognosis.

The clinical staging information primarily drives the initial radiation treatment and prognosis assessment of non-surgical esophageal cancer patients. The 6th edition of the AJCC staging defined abdominal LN as a distant metastasis (15). However, the 7th and 8th editions of the AJCC staging redefined abdominal LN as a regional LN metastasis (6, 16). Numerous studies demonstrated abdominal LN metastasis as a poor prognostic factor (17, 18), but its significance in clinical staging is still unknown. The current study hypothesized that the combination of abdominal LN metastasis and the number of metastatic LNs could be used to develop a revised nodal (rN) staging to improve the disease prediction scheme of ESCC.

## Materials and methods

### Patients

This retrospective study of 707 patients with ESCC receiving definitive (chemo) radiotherapy was conducted at two participating institutes. Eligible patients had a pathological diagnosis of ESCC, completion of (chemo) radiotherapy, and no evidence of distant metastasis imaging before definitive CRT. We excluded patients with multiple primary malignant tumors or survival of less than 3 months after definitive (chemo) radiotherapy. Clinical staging was performed according to the 8th edition of the AJCC TNM staging for ESCC. We extracted complete clinical features of metastatic LNs from pre-treatment imaging examinations, including enhanced diagnostic computed tomography (CT), esophageal ultrasound (EUS), and/or fluorodeoxyglucose-positron emission tomography (FDG-PET). Diagnostic criteria for metastatic LNs on CT include (a) LNs with a short-axis length of at least 10 mm and any node seen in the paraesophageal, tracheoesophageal sulcus, or pericardial angle with a short-axis length of at least 5 mm; (b) LNs with a contrast-enhancing rim or central necrosis. LNs were also considered positive when PET-CT showed a high SUV (except for inflammatory LNs) (19). Endosonographic-directed fine needle aspiration (EUS-FNA) was performed if necessary to minimize the risk of undetected metastatic LNs.

In our study, the abdominal region included the station of paracardial, splenic, common hepatic, left gastric, and celiac nodes. The categorization criteria of nodal variables were defined as follows: number (1–2 LNs; 3–6 LNs; ≥7 LNs) and region (cervical region, thoracic region, and abdominal region). The Medical Ethics Committee of Liaocheng People’s Hospital (2021005) and Shandong Cancer Hospital (SDTHEC20190200) approved the study, and the informed consent was exempted due to the retrospective nature of this study.

### Treatment

All patients in this study received definitive chemo-radiotherapy using intensity-modulated radiotherapy (IMRT) or three-dimensional conformal radiation therapy (3D-CRT). The gross tumor volume (GTV) was contoured based on pre-treatment imaging examinations on the simulation CT scans. The clinical target volumes (CTVs) included the primary tumor, metastatic LNs, and areas at risk of microscopic disease. Patients received a dose of 50–66 Gy/5–6.6 weeks with a conventional fraction. A total of 431 patients received at least one cycle of concurrent chemotherapy. The chemotherapy regimen is a fluorouracil-, paclitaxel-, or platinum-based double-drug combination regimen: fluorouracil (750–1,000 mg/m^2^, continuous intravenous pumping 96 h), cisplatin (75–100 mg/m^2^, intravenous infusion), paclitaxel/paclitaxel liposome (135–175 mg/m^2^, intravenous infusion), or docetaxel (75–100 mg/m^2^, intravenous infusion) repeated every 3–4 weeks.

### Follow-up and statistical analysis

Patients received follow-up every 3 months in the first 2 years and every 6 months after that until death or loss of follow-up. Overall survival (OS) was considered the period from initiation of radiation to the date of last follow-up or mortality, and progression-free survival (PFS) was the duration from treatment date to the date of progression. Fisher’s exact test was used to determine the significance of differences between groups for the region of mLNs. The log-rank test was used for univariate analysis to compare survival of patients with different clinicopathological characteristics. Multivariate Cox proportional hazards regression models were used to evaluate potential associations between survival and clinical factor. A *p-*value less than 0.05 was considered statistically significant. All statistical analyses were performed using the SPSS 23.0 software (SPSS, Inc., Chicago, IL, USA).

## Results

### Baseline characteristics of the patients

A total of 707 ESCC patients, including 529 (75.8%) male and 178 (24.2%) female patients, with a median age of 62 years [range, 21–87 years, most patients were over 60 (72.9%)] were included in this study. A total of 108 (15.3%), 472 (66.8%), and 127 (17.9%) patients were at T2, T3, and T4 stages, respectively. A total of 300 (42.4%) patients were at the N1 stage, making it the most common, followed by the N0 stage with 210 (29.7%) patients, the N2 stage with 170 (24.0%) patients, and the N3 stage with 27 (3.8%) patients. As listed in **Table 1**, the abdominal LN metastasis was correlated with the T category, N category, and tumor location.

**Table 1:** Baseline characteristics of non-surgical patients with no-abdominal LN metastasis vs. abdominal LN metastasis.

Variables	Total (%)	N0 (%)	No-abdominal LN metastasis (%)	Abdominal LN metastasis (%)	*p*
Age (years)					0.530
<60	160 (27.1)	44 (21.0)	91 (22.5)	25 (26.9)	
≥60	547 (72.9)	166 (79.0)	313 (77.5)	68 (73.1)	
Sex					0.592
Male	529 (75.8)	154 (73.3)	305 (75.5)	70 (75.3)	
Female	178 (24.2)	56 (26.7)	99 (24.5)	23 (24.7)	
Smoking					0.651
Yes	352 (49.8)	109 (51.9)	200 (49.5)	43 (46.2)	
No	355 (50.2)	101 (48.1)	204 (50.5)	50 (53.8)	
Drinking					0.242
Yes	277 (39.2)	74 (35.2)	169 (41.8)	34 (36.6)	
No	430 (60.8)	136 (64.8)	235 (58.2)	59 (63.4)	
Tumor location					<0.001
Cervical/Upper	286 (40.4)	85 (40.5)	194 (48.0)	7 (7.5)	
Middle	265 (37.5)	86 (41.0)	142 (35.1)	37 (39.8)	
Lower	156 (22.1)	39 (18.5)	68 (16.8)	49 (52.7)	
8th AJCC T staging					<0.001
T2	108 (15.3)	56 (26.7)	49 (12.1)	3 (3.2)	
T3	472 (66.8)	121 (57.6)	280 (69.3)	71 (76.3)	
T4	127 (17.9)	33 (15.7)	75 (18.6)	19 (20.4)	
8th AJCC N staging					<0.001
N0	210 (29.7)	210 (100)	0	0	
N1	300 (42.4)	0	274 (67.8)	26 (28.0)	
N2	170 (24.0)	0	123 (30.4)	47 (50.5)	
N3	27 (3.8)	0	7 (1.7)	20 (21.5)	
Tumor length					0.175
<5 cm	385 (54.5)	125 (59.5)	214 (53.0)	46 (49.5)	
≥5 cm	322 (45.5)	85 (40.5)	190 (47.0)	47 (50.5)	
Treatment					0.001
CRT	431 (61.0)	106 (50.5)	268 (66.3)	57 (61.3)	
RT alone	276 (39.0)	104 (49.5)	136 (33.7)	36 (38.9)	

LNs, lymph nodes; CRT, chemoradiotherapy; RT, radiotherapy.

### Abdominal LN metastasis: prognostic significance

**Figure 1:**
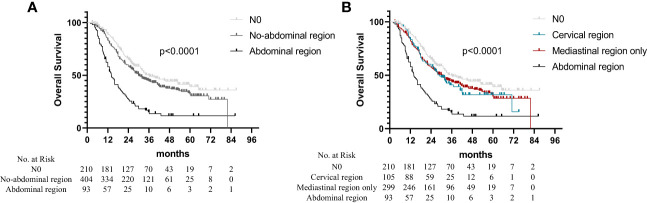
Overall survival in non-surgical esophageal squamous cell carcinoma patients according to different regions of metastatic lymph nodes. **(A)** Based on the region of LN metastasis. **(B)** The patients without abdominal LN metastasis were subdivided into cervical LNs metastasis and non-cervical LN metastasis (mediastinal LNs metastasis only).

The median OS times were 36.0 months (95% CI: 26.8–45.3 months), 30.0 months (95% CI: 25.6–34.4 months), and 14.0 months (95% CI: 11.5–16.5 months) for the patients with N0, non-abdominal LN metastasis, and abdominal LN metastasis, respectively. The OS of the patients with abdominal LN metastasis was significantly worse than that of the other regional metastases (**Figure 1A**; *p* < 0.001). Based on the region of LN metastasis, the patients without abdominal LN metastasis were subdivided into cervical LN metastasis and non-cervical LN metastasis (mediastinal LN metastasis only). However, there was no difference in the OS times between the patients with cervical LN metastasis and non-cervical LN metastasis in the non-abdominal LN metastasis subgroups (**Figure 1B**; *p* = 0.674). The multivariate analysis identified abdominal LN metastasis and the number of metastatic LNs as independent prognostic factors for the OS time of patients (**Table 2**).

**Table 2:** Univariate and multivariable Cox regression analyses for OS of 707 ESCC patients.

Variables	Univariate analysis			Multivariate analysis		
	HR	95% CI	*p*	HR	95% CI	*p*
Age (baseline: <60)	1					
≥60	1.095	0.868–1.382	0.442			
Sex (baseline: Male)	1					
Female	0.803	0.659–0.977	0.029			NS
Smoking (baseline: No)	1					
Yes	1.215	1.001–1.475	0.049			NS
Drinking (baseline: No)	1					
Yes	1.237	1.018–1.503	0.033	1.331	1.091–1.623	0.005
Tumor location (baseline: Cervical/Upper)	1			1		
Middle	1.580	1.257–1.986	<0.001	1.410	1.116–1.780	0.004
Lower	2.417	1.880–3.108	<0.001	1.823	1.386–2.396	<0.001
T staging (baseline: T1/2)						
T3	1.568	1.162–2.117	0.003	1.225	0.889–1.670	0.198
T4	2.342	1.661–3.102	<0.001	1.843	1.292–2.629	0.001
Tumor length (baseline: <5 cm)	1					
≥5 cm	1.234	1.017–1.498	0.033			NS
Treatment (baseline: RT alone)	1					
CRT	0.757	0.623–0.920	0.005	0.679	0.554–0.833	<0.001
N staging (baseline: N0)	1			1		
N1	1.119	0.875–1.432	0.372	1.078	.835–1.390	0.565
N2	2.037	1.567–2.647	<0.001	1.770	1.330–2.354	<0.001
N3	4.032	2.560–6.352	<0.001	2.694	1.609–4.509	<0.001
LN location (baseline: N0/no-abdominal region)	1			1		
abdominal region	2.561	1.998–3.284	<0.001	1.518	1.120–2.058	0.007

PFS, progression-free survival; OS, overall survival; HR, hazard ratio; CI, confidence interval; CRT, chemoradiotherapy; RT, radiotherapy; NS, no significance.

### Different combinations of the LNs variables: N staging strategy

**Figure 2:**
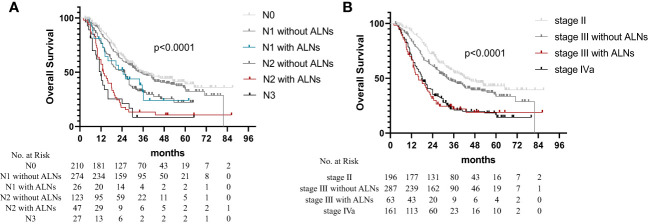
Overall survival in non-surgical esophageal squamous cell carcinoma patients according to the 8th AJCC staging. **(A)** N1 and N2 patients were divided into four groups as follows: N1 disease without abdominal LN metastasis (N1R1), N1 disease with abdominal LN metastasis (N1R2), N2 disease without abdominal LN metastasis (N2R1), and N2 disease with abdominal LN metastasis (N2R2). **(B)** Patients with stage III were divided into stage III disease without abdominal LN metastasis and stage III disease with abdominal LN metastasis.

Based on the number of metastatic LNs and abdominal LN metastasis, different combinations of LN variables were further analyzed to explore their prognostic potential. The node variables were categorized as follows: based on the number of metastatic LNs (N1: 1–2 LNs; N2: 3–6 LNs; and N3: ≥7 LNs) and based on the region involved (R1: no-abdominal LN metastasis; and R2: abdominal LN metastasis). All patients were divided into six groups as follows: N0, N1 without abdominal LN metastasis (N1R1), N1 with abdominal LN metastasis (N1R2), N2 without abdominal LN metastasis (N2R1), N2 with abdominal LN metastasis (N2R2), and N3 (N3R1-2). The survival curve observed a significant difference in OS (**Figure 2A**; *p* < 0.001), with worse survival occurring in patients with abdominal LN metastasis. We found no significant difference in OS between N1R2 subsets and N2R1 subsets (*p* = 0.994). The OS of N2R2 patients is not significantly different from the patients with N3 (*p* = 0.316).

The patients with clinical stage II ESCC had no abdominal LN metastasis. Based on the clinical staging system, all the patients were divided into four stages: stage II, stage III without abdominal LN metastasis, stage III with abdominal LN metastasis, and stage IVa. The OS times of the patients with stage III with abdominal LN metastasis and patients with stage IVa showed no statistically significant difference, as shown in **Figure 2B** (*p* = 0.629). The multivariate analysis showed similar hazard ratios (HRs) of the N3R1-2 (HR = 2.746) and N1-2R2 (HR = 2.322) groups (**Table 3**). Therefore, the N1R1 subset was defined as rN1, the N2R1 subset was defined as rN2, and the N3R1-2 and N1-2R2 subsets were defined as rN3.

**Table 3:** Effect of different N subsets on OS of 707 patients with ESCC in multivariable analyses.

	HR	95% CI	*p*
N0	1		
N_1_R_1_	1.145	0.885–1.481	0.303
N_2_R_1_	1.755	1.303–2.364	<0.001
N_1-2_R_2_	2.322	1.729–3.131	<0.001
N_3_R_1-2_	2.746	1.693–4.185	<0.001

PFS, progression-free survival; OS, overall survival; HR, hazard ratio; CI, confidence interval.

Multivariable analyses adjusted for age, smoking, drinking, T category, treatment, tumor length, and tumor location.

**Figure 3:**
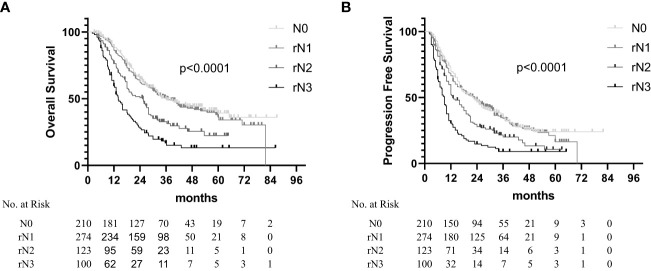
Overall survival **(A)** and progression-free survival **(B)** in non-surgical esophageal squamous cell carcinoma patients according to the rN staging.

The results showed that the OS and PFS times were significantly correlated with the rN staging. The median OS times were 35.7 months (95% CI, 29.2–42.2 months), 25.2 months (95% CI, 19.2–31.2 months), and 14.0 months (95% CI, 11.8–16.2 months) for the rN1, rN2, and rN3 subsets, respectively (**Figure 3A**, *p* < 0.001). The median PFS times were 22.0 months (95% CI, 17.1–26.0 months), 23.0 months (95% CI, 18.3–27.2 months), 13.0 months (95% CI, 10.1–15.9 months), and 9.0 months (95% CI, 7.9–10.1 months) for the N0, rN1, rN2, and rN3 subsets, respectively (**Figure 3B**, *p* < 0.001). The multivariate analysis identified rN staging as an independent factor for predicting the PFS and OS (**Table 4**). When the patients with abdominal LN metastasis were upstaged to rN3, the distribution of rN staging subsets tended to be justified [rN3: 14.1% (100/707) vs. AJCC N3: 3.8% (27/707)]. The survival analysis results stratified by the rN stage, 8th AJCC N stage, and 11th Japan Esophagus Society (JES) N stage are listed in **Table 5**. The comparison of prognostic performance based on the stratification of each staging system showed that the rN stage could differentiate in the OS time of each subgroup based on the 8th AJCC N staging or the 11th JES N staging (**Supplementary Figures 1**, **2**). However, the other staging systems could not differentiate in the OS time of each subgroup based on the rN staging (**Supplementary Figures 3**, **4**).

**Table 4:** Effect of rN staging on OS of 707 patients with ESCC in multivariable analyses.

	PFS			OS		
	HR	95% CI	*p*	HR	95% CI	*p*
N0	1		<0.001	1		<0.001
rN1	1.109	0.888–1.384	0.361	1.142	0.883–1.478	0.312
rN2	1.623	1.250–2.109	<0.001	1.745	1.296–2.351	<0.001
rN3	2.385	1.799–3.162	<0.001	2.454	1.793–3.358	<0.001

PFS, progression-free survival; OS, overall survival; HR, hazard ratio; CI, confidence interval.

Multivariable analyses adjusted for age, smoking, drinking, T category, treatment, tumor length, and tumor location.

**Table 5:** Distribution and survival of AJCC N stage and JES N stage based on different rN stages.

	N0		rN1		rN2		rN3		
	NO.	M.OS (M)	NO.	M.OS (M)	NO.	M.OS (M)	NO.	M.OS (M)	*p*
8th AJCC cN staging									
N0	210 (100%)	36.0							
N1			274 (91.3%)	35.5			26 (8.7%)	24.5	0.059
N2					123 (72.4%)	25.2	47 (27.6%)	13.5	0.001
N3							27 (100%)	12.0	
*p*								0.025	
11th JES N staging									
N0	210 (100%)	36.0							
N1			119 (84.4%)	38.0	4 (2.8%)	15.0	18 (12.8%)	22.2	0.109
N2			130 (50.0%)	36.0	89 (34.2%)	25.6	41 (15.8%)	13.0	<0.001
N3			25 (26.0%)	30.2	30 (31.3)	19.0	41 (42.7%)	13.5	0.015
*p*				0.458		0.883		0.172	

NO., number; M.OS(M), Median overall survival (months).

## Discussion

According to the 8th edition of the Cancer Staging Manual of Worldwide Esophageal Cancer Collaboration (WECC), 60% of ESCC patients have LN metastasis, while the N3 patients account for only 2.3% of the patients with LN metastasis (7). This disproportionate ratio might affect staging accuracy and lead to missing the high-risk patients. The diagnosis of metastatic LNs in non-surgical patients is primarily based on physical examination, endoscopy, and imaging examination. Although cN staging, ypN staging, and pN staging systems have similar staging criteria, the similarity in their prognostic significance is uncertain. This study analyzed the effects of a combination of LN variables on the survival of patients and developed an rN staging system based on the number of metastatic LNs and abdominal LN metastasis, showing high accuracy in predicting the prognosis of ESCC after definitive (chemo) radiotherapy.

Recent studies reported that the N stage could not clearly distinguish between the prognostic risks of different patients. Based on a dataset of 8,156 clinically staged ESCC patients from the WECC, Rice et al. reported that the patients with cN0 could not be distinguished from those with cN1 (7). In addition, Yamasaki et al. observed 665 ESCC patients who underwent esophagectomy and reported no significant differences in the survival times of patients with N2 and N3 (8). These results suggested that some high-risk patients might be hidden among the N2 patients, and some low-risk patients among the N1 patients might have a similar prognosis to that of N0 patients. Therefore, this should be further clarified by incorporating more LN metastasis factors.

Based on anatomic regions, the AJCC staging system for ESCC divided the esophageal lymphatic drainage into three groups, namely, cervical, thoracic, and abdominal (16). The 6th edition of the AJCC staging system defined abdominal LN metastasis as the M1 stage (15). However, the 7th AJCC staging system redefined abdominal LN metastasis as regional LN metastasis (16). Rutegard et al. retrospectively analyzed 446 surgical EC patients and reported that the patients with celiac LN metastasis had a 52% increased risk of disease-specific mortality (17). Among the ESCC patients receiving CRT, Trovo et al. reported that the patients with celiac LN metastasis had worse survival times (18). The current study found that the patients with the same N stage and clinical stage with abdominal LN metastasis had a worse prognosis than those without abdominal LN metastasis. Moreover, the multivariate analysis identified abdominal LN metastasis as an independent prognostic factor. This suggested that the abdominal LN metastasis might be beneficial for improving the prediction accuracy of N staging. Therefore, this study included abdominal LN metastasis in the rN staging system.

Owing to their specific anatomical location, the local salvage therapy for the patients with recurrence supraclavicular LN metastasis might mitigate its adverse effects on survival. Yano et al. (20) explored the effects of salvage therapy on 35 patients with cervical LN metastasis recurrence after surgery, who mainly received local therapies, such as radiotherapy or surgery. They reported that the salvage local therapy had a survival benefit in the event of a single-node recurrence in the neck. Watanabe et al. (21) studied 33 patients with recurrent supraclavicular LN metastasis who underwent LN dissection and adjuvant chemotherapy after surgery. With a median follow-up period of 54 months, all the patients survived. Therefore, local salvage therapy is regarded as a safe and effective method for the treatment of cervical/supraclavicular LN metastasis recurrence. Numerous studies reported that supraclavicular LN metastasis was not an independent prognostic factor after surgery for thoracic ESCC (22–24). The feasibility of salvage treatment after relapse in this region might be one of the reasons for its better prognosis compared to that in the other regions.

Previous studies showed that the impact of abdominal LN metastasis on the survival of patients was related to the location of the primary tumor (25). The current JES N staging system is also based on the location of the primary tumor and LN metastasis (26). The tumor’s location can affect the prognosis of surgical patients and is included in the pathological staging system. Although the impact of tumor location on the prognosis of surgical patients is still controversial, the studies showed that the tumor in the lower segment had a better prognosis as compared to those in the middle and upper segments, especially among the patients with T3N0M0 (27–30). Interestingly, this study identified the primary tumor location as an independent prognostic factor for the patients receiving radiotherapy; these results were consistent with previous studies (31, 32). Therefore, the primary tumor location alone could be used as a prognostic stratification factor. This study included two variables for LN metastasis to develop a new staging system, which could better differentiate the prognosis of patients in the 11th JES N subgroup. However, this staging strategy should be verified and optimized using large samples and multicenter data.

There were certain limitations to this study. First, there might be a bias in the patient’s treatment plans, such as the target volume and dose of radiotherapy. Salvage therapy after failure was also an essential factor that could affect the survival of patients. Second, the accuracy of imaging-based diagnosis of positive LNs might also be biased, especially the number of metastatic LNs, which was a major intention of this study for including more factors to optimize the N staging. Finally, this study only included standard clinicopathological features and lacked relevant information, such as the degree of pathological differentiation.

## Conclusions

This study established a novel N staging system using the number of metastatic LNs and abdominal LN metastasis. The staging showed good accuracy and stratification ability and was superior to the other N staging systems, such as AJCC and JES N staging system with a single metastatic variable. Moreover, this staging was a potential prognostic indicator for the patients with ESCC who received definitive radiotherapy. However, the sample size of this study was relatively small, needing further verification and improvement.

## Data availability statement

The original contributions presented in the study are included in the article/**Supplementary Material**. Further inquiries can be directed to the corresponding author.

## Ethics statement

The study was approved by the Medical Ethics Committee of Liaocheng People’s Hospital (2021005) and Shandong Cancer Hospital (SDTHEC20190200). The studies were conducted in accordance with the local legislation and institutional requirements. Written informed consent for participation was not required from the participants or the participants’ legal guardians/next of kin in accordance with the national legislation and institutional requirements.

## Author contributions

ZZ: conceptualization, formal analysis, investigation, resources, writing—original draft, and approval of the manuscript. HW: conceptualization, data curation, formal analysis, methodology, visualization, writing—original draft, and approval of the manuscript. YL: formal analysis, methodology, and approval of the manuscript. MHL: data curation, investigation, resources, and approval of the manuscript. MJL: conceptualization, data curation, methodology, supervision, writing—original draft, writing—review and editing, and approval of the manuscript. All authors contributed to the article and approved the submitted version.
