# Supplementary figures and images for: Abdominal lymph node metastasis in non-surgical esophageal squamous cell carcinoma: prognostic significance and a novel staging strategy
Source: Front Oncol. 2023 Oct 9;13:1234426. doi: 10.3389/fonc.2023.1234426 (PMC10591313; doi:10.3389/fonc.2023.1234426)

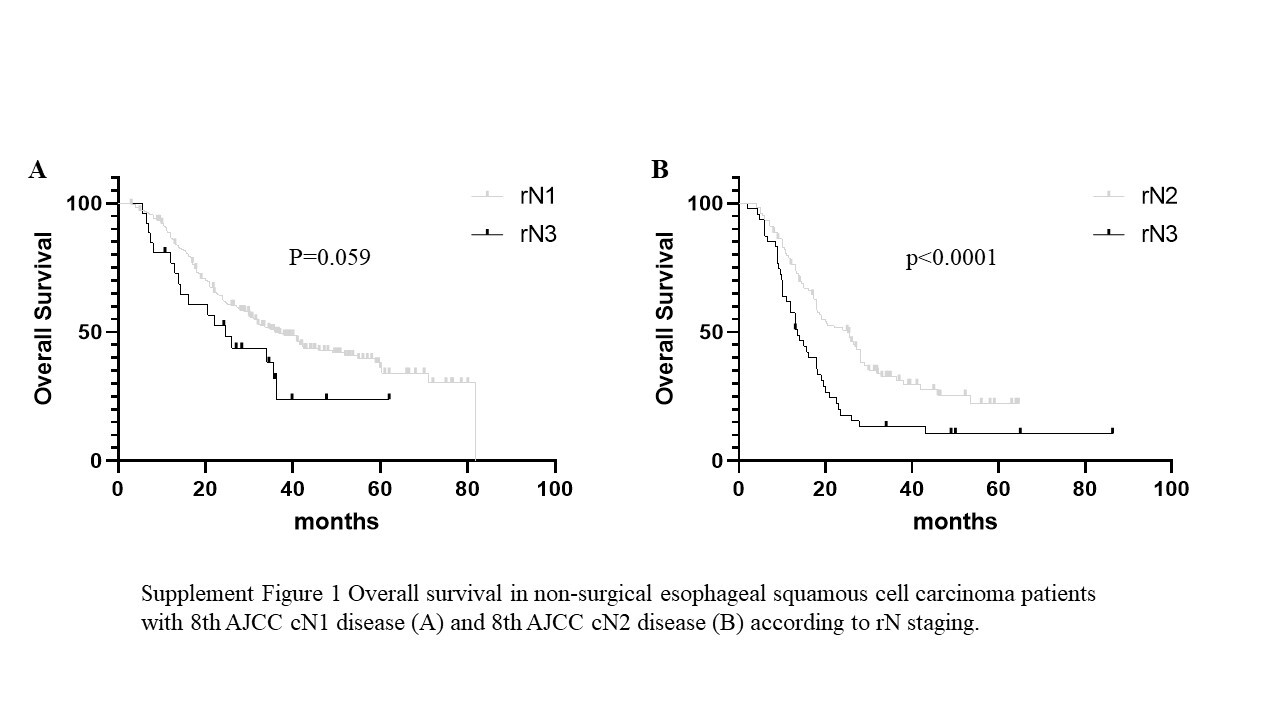

Supplement: Supplementary file 1 [file Image_1.jpeg]

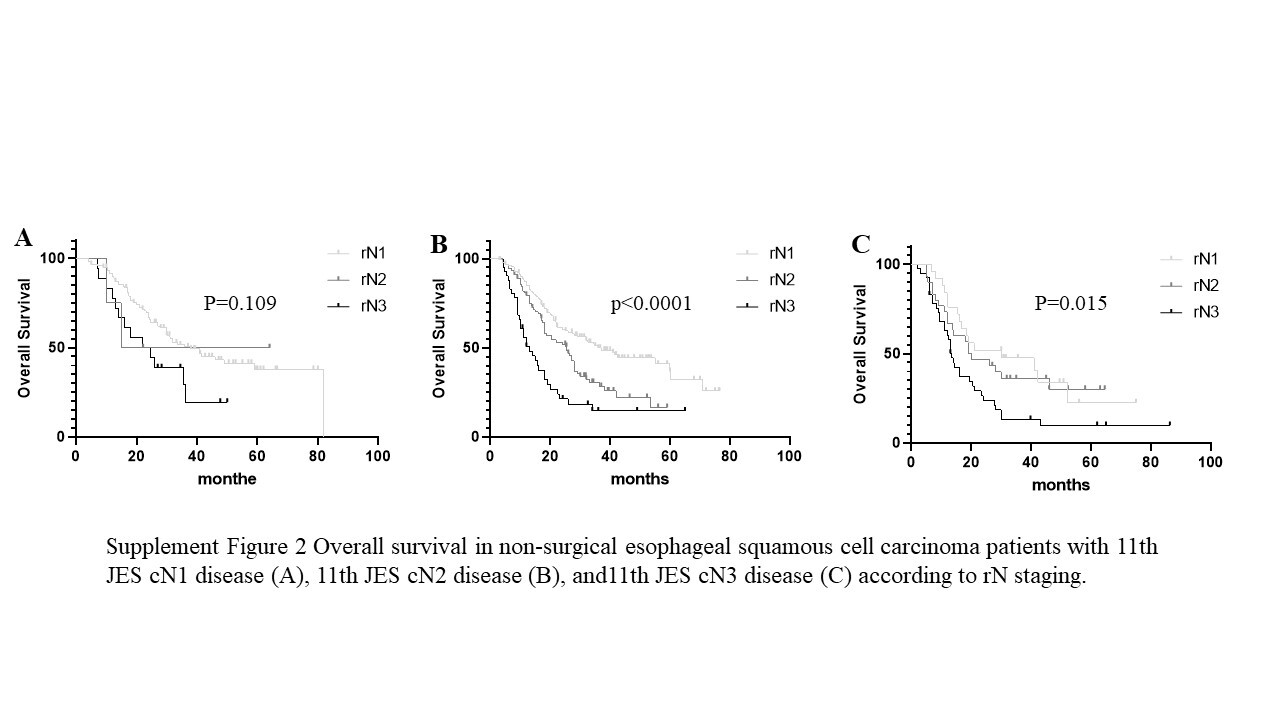

Supplement: Supplementary file 2 [file Image_2.jpeg]

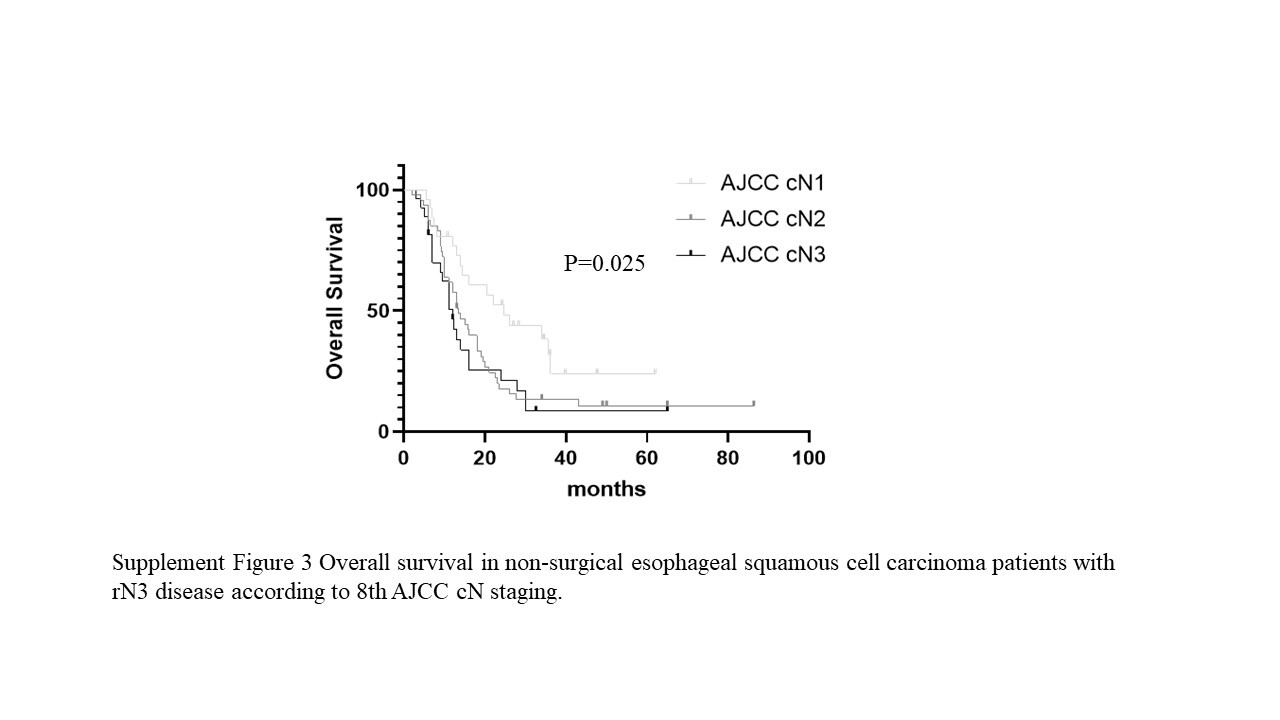

Supplement: Supplementary file 3 [file Image_3.jpeg]

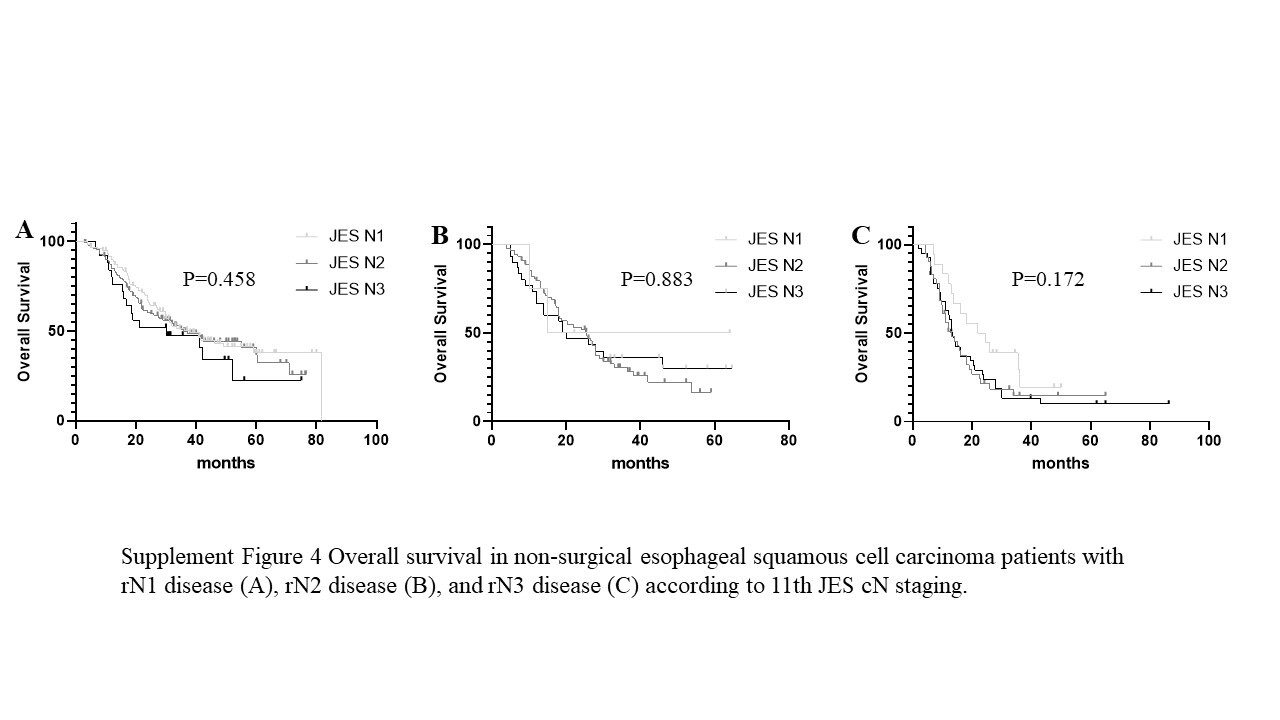

Supplement: Supplementary file 4 [file Image_4.jpeg]
